# Prevalence and risk factors for self-reported non-communicable diseases among older Ugandans: a cross-sectional study

**DOI:** 10.3402/gha.v8.27923

**Published:** 2015-07-22

**Authors:** Stephen Ojiambo Wandera, Betty Kwagala, James Ntozi

**Affiliations:** Department of Population Studies, School of Statistics and Planning, College of Business and Management Sciences, Makerere University, Kampala, Uganda

**Keywords:** Africa, Uganda, chronic diseases, non-communicable diseases, elderly

## Abstract

**Background:**

There is limited evidence about the prevalence and risk factors for non-communicable diseases (NCDs) among older Ugandans. Therefore, this article is aimed at investigating the prevalence of self-reported NCDs and their associated risk factors using a nationally representative sample.

**Design:**

We conducted a secondary analysis of the 2010 Uganda National Household Survey (UNHS) using a weighted sample of 2,382 older people. Frequency distributions for descriptive statistics and Pearson chi-square tests to identify the association between self-reported NCDs and selected explanatory variables were done. Finally, multivariable complementary log–log regressions to estimate the risk factors for self-reported NCDs among older people in Uganda were done.

**Results:**

About 2 in 10 (23%) older persons reported at least one NCD [including hypertension (16%), diabetes (3%), and heart disease (9%)]. Among all older people, reporting NCDs was higher among those aged 60–69 and 70–79; Muslims; and Pentecostals and Seventh Day Adventists (SDAs). In addition, the likelihood of reporting NCDs was higher among older persons who depended on remittances and earned wages; owned a bicycle; were sick in the last 30 days; were disabled; and were women. Conversely, the odds of reporting NCDs were lower for those who were relatives of household heads and were poor.

**Conclusions:**

In Uganda, self-reported NCDs were associated with advanced age, being a woman, having a disability, ill health in the past 30 days, being rich, depended on remittances and earning wages, being Muslim, Pentecostal and SDAs, and household headship. The Ministry of Health should prevent and manage NCDs by creating awareness in the public and improving the supply of essential drugs for these health conditions. Finally, there is a need for specialised surveillance studies of older people to monitor the trends and patterns of NCDs over time.

Non-communicable diseases (NCDs) were the leading cause of mortality, morbidity, and disability globally in 2014 (1, 2). According to the World Health Organization (WHO), NCDs account for 38 million deaths in the world each year (3). About three quarters (28 million) of these deaths occur in low- and middle-income countries. NCDs associated deaths are projected to increase by 15% between 2010 and 2020, and 20% globally and in Africa (3, 4). In South Africa, the prevalence of NCDs was 50% (4). The four commonly reported NCDs worldwide are cardiovascular diseases (heart attacks and stroke), followed by cancers, chronic respiratory diseases, and diabetes (4–6).

Uganda like other developing countries is experiencing an epidemiological transition characterised by an increasing burden of NCDs and population ageing (7–10). The rise in the prevalence of NCDs has resulted in a double burden of diseases – both communicable and NCDs – in developing countries including Uganda (11, 12). The population of older persons is steadily increasing (9). In Uganda, the population of older persons increased from 1.1 million in 2002 to 1.3 million in 2010 (13) and is expected to increase from 1.6 million in 2014 to 5.5 million by 2050 (14). Uganda's Ministry of Health has initiated measures to address NCDs through policy and health programmes aimed at prevention and management (11).

There are about five commonly known risk factors for NCDs namely, physical inactivity, low intake of fruits and vegetables, overweight or obesity, smoking, and alcohol consumption (4, 5, 7, 10, 15, 16). These factors can be categorised into two factors – lifestyle or behavioural factors and biological factors (16). Sedentary life style or physical inactivity has been associated with NCDs in South Africa (4) and Ghana (7). Overweight or obesity is associated with NCDs in Ghana (7). These modifiable lifestyle factors operate in the context of demographic and socio-economic factors.

Socio-economic and demographic factors have been associated with NCDs. Being female or male, advanced age, lack of formal education, greater wealth, and residence in urban areas were associated with NCDs in South Africa (4) and Ghana (7). With respect to marital status, loss of spouse (widowhood) or separation from spouse is a key risk factor for NCDs (4, 7). Occupational risk factors are the important predictors of NCDs. One's occupation and place of work can cause or exacerbate NCDs (6), for example, sedentary work, work stress, exposure to carcinogens, shift work, and unsupportive environments such as provision of unhealthy food at workplaces.

There is limited evidence about the prevalence and risk factors for chronic NCDs among older Ugandans, using a nationally representative sample. Therefore, this article investigated the prevalence of self-reported NCDs and their associated risk factors.

## Methods

### Data source

We used the 2010 Uganda National Household Survey (UNHS) data with the permission from the Uganda Bureau of Statistics (UBOS) during the data sharing and dissemination workshop at the College of Business and Management Sciences (COBAMS) in 2012. The UNHS used a two-stage stratified sampling. At the first stage, 712 enumeration areas were drawn using probability proportional to size. At the second stage, households were drawn using systematic sampling. A total of 6,800 households were interviewed in the survey (17). Older persons were selected from the sample using the variable age. Persons aged 50 years and older were selected for further analysis, forming a weighted sample of 2,382 older people. The decision to select persons aged 50 years and above was based on the recommendation by WHO and the INDEPTH network studies, which define older persons starting at age 50 for African contexts (18, 19). This research was conducted with the ethical approval (number: SS 3198) of the Uganda National Council of Science and Technology (UNCST) and the research and higher degrees committee of the School of Statistics and Applied Economics of Makerere University.

### Outcome variable

The outcome variable was self-reported prevalence of NCDs among older people. In the UNHS, respondents were asked to report whether they had any one of the three NCDs: hypertension, diabetes, or heart disease. The questions allowed multiple responses to these three health conditions. These responses were coded as binary (0 =did not report any NCDs and 1 =reported at least one of the three NCDs). Thus, self-reported NCDs meant the reporting of diabetes, heart disease, and hypertension, recoded as binary variable (17).

### Explanatory variables

Several demographic, socio-economic, and health variables covered in the UNHS survey were included in the analyses as risk factors for self-reported NCDs. Demographic factors included: gender (male or female), age group, region, place of residence (rural or urban), living arrangement, relationship to household head, and marital status. Age was recoded into four age categories: 50–59, 60–69, 70–79, and 80+. Region had four categories (1=central, 2=eastern, 3=northern, and 4=western). Living arrangements were recoded into two categories (living alone and with others). Relationship to household head was recoded into three categories (1=head, 2=spouse, and 3=relative). Marital status was recoded into three categories (1=married, 2=separated or divorced or never married, and 3=widowed). Three never married older persons were merged with the separated and divorced category in the data because they were very few.

Socio-economic factors included: education level, religion, household poverty status, household major source of earnings, learning a technical skill, and ownership of bicycle. Education level was recoded into no education, primary and secondary, or higher education. Religion was recoded into Catholic, Anglican, Muslim, Pentecostal and Seventh Day Adventists (SDAs), and others. Household poverty status was generated from household expenditures and recoded (1=poor if a household spent less than $1 a day and 0=not poor, if a household spent greater than $1 a day). Household major source of earnings was recoded into farming, wages, and remittances. Learning a technical skill and household bicycle ownership were binary (0=no, 1=yes).

Health-related information was collected on illnesses in the last 30 days preceding the survey (17). Ill health was a binary variable (0=not sick, 1=sick). Disability was measured by asking six questions on functional limitations on both activities of daily living (ADLs) and instrumental activities of daily living (IADLs). ADLs, which mainly focused on body impairments, included difficulties in seeing, hearing, walking, and concentrating or remembering. IADLs, which relate to personal care, were measured using difficulties with washing or bathing, feeding, dressing, and toileting. Respondents were asked whether they had difficulty:

1. Seing, even if he/she is wearing glasses?
2. Hearing, even if he/she is wearing a hearing aid?
3. Walking or climbing steps?
4. Remembering or concentrating?
5. With self-care such as washing all over or dressing, feeding, and toileting?
6. Communicating – for example, understanding others or others understanding him/her, because of a physical, mental, or emotional health condition?

These six questions were originally coded into five categories (1=No, no difficulty, 2=Yes – some difficulty, 3=Yes – a lot of difficulty, 4=cannot perform at all, and 8=don't know). Among the older persons, there was only one respondent who did not know that he or she had sight disability. Disability or being disabled was defined or interpreted as either (1) having a lot of difficulty on any of the six indicators; (2) being unable to perform at all on any of the six indicators; or (3) having some difficulty with at least two of the six indicators. This measurement of disability has been used in other studies (20–22). Using these three aspects is better than using ‘having some difficulty on any indicator’ to measure disability (23).

### Statistical analyses

We conducted Pearson chi-square tests and multivariable complementary log–log regressions for the prevalence and correlates of NCDs, respectively. We included variables that were significantly (*p*<0.05) associated with NCDs for all older persons irrespective of whether that was consistent among men or women only. We used complementary log–log regression to estimate the odds of reporting NCDs among older persons in Uganda. The complementary log–log regression model is recommended for rare outcomes or when the data are asymmetrical (24–26) and the outcome is binary. In this case, NCDs had an uneven distribution of 23% in the population of older adults. We applied gender segregation to the analysis. We used STATA version 13 during the analysis. We used survey weights to account for the complex survey design including clustering and stratification.

## Results

### Prevalence of NCDs

Table 1 shows the association of NCDs by gender among older people in Uganda. Overall, 2 in 10 older people (23%) reported NCDs. There were significant differences in the reporting of NCDs between men and women – older men had a lower prevalence of NCDs than older women did (16% vs. 30%; *p*<0.01). For all individual variables, this pattern was consistent. Older persons reported hypertension (16%) more than heart disease (9%) and diabetes (3%). Older women reported hypertension (20% vs. 10%; *p*<0.01) and heart diseases (12% vs. 6%; *p*<0.01) more than the men were. There were no significant differences between men and women on reporting of diabetes (Table 1).

**Table 1:** NCDs by type and gender

	Men	Women	All	
				
Variables	Number (%)	Number (%)	Number (%)	*p*
Reported diabetes				0.67
No	1,099 (96.7)	1,209 (97.0)	2,308 (96.9)	
Yes	38 (3.3)	37 (3.0)	74 (3.1)	
Reported hypertension				<0.01
No	1,013 (89.2)	993 (79.7)	2,006 (84.2)	
Yes	123 (10.8)	253 (20.3)	376 (15.8)	
Reported heart diseases				<0.01
No	1,071 (94.3)	1,098 (88.1)	2,169 (91.1)	
Yes	65 (5.7)	148 (11.9)	213 (8.9)	
Number of NCDs reported				<0.01
0	949 (83.6)	878 (70.4)	1,827 (76.7)	
1	153 (13.4)	305 (24.5)	458 (19.2)	
2	30 (2.6)	56 (4.5)	86 (3.6)	
3	4 (0.4)	7 (0.6)	11 (0.5)	<0.01
Reported at least one NCD				
No	949 (83.6)	878 (70.4)	1,827 (76.7)	
Yes	187 (16.4)	368 (29.6)	555 (23.3)	
Total	1,136 (100.0)	1,246 (100.0)	2,382 (100.0)	

### Association between NCDs and demographic and socio-economic factors

Table 2 shows the association between reporting at least one of the three NCDs and demographic, socio-economic, and health-related factors among older people in Uganda. We stratified the analyses by gender. Two in ten (23%) older persons reported at least one NCD, and the prevalence significantly varied between men and women (16% vs. 30%; *p*<0.01).

**Table 2:** Association between self-reported NCDs and risk factors stratified by gender among older people in Uganda

	Men		Women		All	
			
Variables	Number (% NCDs)	*p*	Number (% NCDs)	*p*	Number (% NCDs)	*p*
Gender						<0.01
Women					1,246 (29.6)	
Men					1,136 (16.4)	
Age group		0.11		0.01		<0.01
50–59	524 (14.6)		542 (23.8)		1,066 (19.3)	
60–69	313 (14.9)		356 (34.7)		670 (25.4)	
70–79	206 (21.7)		228 (34.7)		433 (28.5)	
80+	94 (20.1)		120 (30.6)		213 (26.0)	
Region		0.29		<0.01		<0.01
Central	278 (17.8)		311 (35.2)		589 (27.0)	
Eastern	371 (18.8)		358 (33.7)		728 (26.1)	
Northern	216 (13.0)		253 (21.8)		470 (17.7)	
Western	271 (14.6)		324 (25.7)		595 (20.6)	
Place of residence		0.32		0.19		0.11
Rural	1,032 (16.0)		1,131 (28.8)		2,162 (22.7)	
Urban	104 (20.4)		115 (36.5)		220 (28.8)	
Living alone		0.35		0.47		0.80
No	1,022 (16.8)		1,145 (29.3)		2,167 (23.4)	
Yes	114 (13.4)		101 (32.9)		215 (22.6)	
Relationship to household head		0.85		<0.01		0.26
Head	995 (16.2)		669 (34.0)		1,664 (23.3)	
Spouse	72 (17.9)		387 (26.7)		458 (25.3)	
Relative	69 (19.0)		191 (19.7)		260 (19.5)	
Marital status		0.39		0.19		<0.01
Married	919 (16.5)		477 (26.4)		1,396 (19.9)	
Divorced/separated/never married	84 (11.8)		158 (29.4)		242 (23.3)	
Widowed	133 (19.1)		611 (32.0)		744 (29.7)	
Education level		0.94		0.54		0.27
None	713 (16.7)		908 (30.3)		1,621 (24.3)	
Primary	302 (15.9)		287 (26.8)		589 (21.2)	
Secondary +	121 (15.8)		51 (31.3)		172 (21.0)	
Religion		0.01		<0.01		<0.01
Catholic	527 (14.2)		549 (25.0)		1,076 (19.7)	
Anglican	398 (14.1)		448 (28.6)		846 (21.8)	
Muslim	110 (29.4)		96 (49.0)		206 (38.6)	
Pentecostal	56 (17.9)		108 (40.0)		164 (32.5)	
SDA and others	45 (29.1)		44 (27.3)		90 (28.2)	
Poverty status		0.24		0.01		0.01
Non-poor	883 (17.1)		955 (31.8)		1,838 (24.7)	
Poor	253 (14.0)		291 (22.2)		544 (18.4)	
Major source of earnings		0.05		0.01		<0.01
Farming	720 (14.6)		731 (26.5)		1,451 (20.6)	
Wages	330 (18.1)		307 (30.7)		637 (24.2)	
Remittances	86 (25.1)		208 (38.7)		294 (34.7)	
Learnt a trade or technical skill		0.06		<0.01		<0.01
No	875 (15.2)		983 (27.0)		1,858 (21.5)	
Yes	261 (20.5)		263 (39.0)		524 (29.8)	
Household owns bicycle		0.01		0.72		0.78
No	590 (13.3)		846 (29.9)		1,436 (23.1)	
Yes	546 (19.8)		400 (28.8)		946 (23.6)	
Was ill or injured during past 30 days		0.02		<0.01		<0.01
No	495 (13.1)		409 (18.7)		904 (15.6)	
Yes	641 (19.0)		837 (34.9)		1,478 (28.0)	
Ever smoked/smokes		0.01		0.60		<0.01
No	775 (18.4)		1,042 (29.9)		1,817 (25.0)	
Yes	361 (12.2)		204 (27.8)		565 (17.8)	
Disabled		<0.01		<0.01		<0.01
No	822 (12.8)		878 (24.6)		1,600 (18.5)	
Yes	314 (25.9)		368 (37.9)		782 (33.1)	
Total	1,136 (16.4)		1,246 (29.6)		2,382 (23.3)	

Among all older people, reporting at least one NCD varied significantly by gender, age group, region, marital status, religion, poverty status, source of earnings, having a technical skill, owning a bicycle, being sick in the last 30 days, smoking, and disability (*p*<0.02). Reporting an NCD was highest among women (30%), those aged 70–79 (29%), and from central region (27%). Older persons who were widowed (30%), Muslims (39%), not poor (25%), depended on remittances (35%), and had a technical skill (30%) reported higher prevalence of disability. Finally, among older people, reporting an NCD was highest among those who were sick (28%) and those who were disabled (33%). Self-reported NCDs did not vary significantly by place of residence (*p*=0.11), living arrangement (*p*=0.80), household headship (*p*=0.26), education level (*p*=0.27), and bicycle ownership (*p*=0.78) among older people.

Among older men, the factors associated with reporting an NCD were religion (*p*=0.01), household bicycle ownership (*p*=0.01), being sick in the past 30 days (*p*=0.02), smoking (*p*=0.01), and being disabled (*p*<0.01). NCDs were highly reported among older men who were Muslims (29%), owned a bicycle (20%), were sick (19%), and reported disability (26%). Self-reported NCDs did not vary significantly by age group (*p*=0.11), region (*p*=0.29), residence (*p*=0.32), living alone (*p*=0.35), relationship to household headship (*p*=0.85), marital status (*p*=0.39), educational level (*p*=0.94), poverty status (*p*=0.24), source of earnings (*p*=0.05), and having a technical skill (*p*=0.06).

Among older women, reporting of NCDs was associated with age group (*p*=0.01), region (*p*<0.01), relationship to household head (*p*<0.01), religion (*p*<0.01), poverty status (*p*=0.01), source of earnings (*p*=0.01), having learnt a technical skill (*p*<0.01), being sick in the past 30 days (*p*<0.01), and being disabled (*p*<0.01). The prevalence of self-reported NCDs among older women was highest among those aged 70–79 (29%), in central region (35%), headed households (34%), Muslims (49%), and those in non-poor households (32%). In addition, NCDs were more prevalent among women who depended on remittances (39%), had a technical skill (39%), were ill or sick in the last 30 days (35%), and were disabled (38%). Reporting of an NCD among older women did not vary significantly by place of residence (*p*=0.19), living alone (*p*=0.47), educational level (*p*=0.54), bicycle ownership (*p*=0.72), and smoking (*p*=0.60).

### Multivariable analysis

Table 3 shows the results of multivariable complementary log–log regression of factors associated with reporting NCDs among older persons stratified by gender.

**Table 3:** Adjusted odds ratios from complementary log–log regression of NCDs among older people in Uganda

	Men		Women		All	
			
	OR	95% CI	OR	95% CI	OR	95% CI
Age group						
50–59	1		1		1	
60–69	1.03	(0.69–1.54)	1.44*	(1.09–1.92)	1.30*	(1.03–1.63)
70–79	1.30	(0.87–1.95)	1.36	(0.97–1.89)	1.32*	(1.02–1.72)
80+	1.10	(0.64–1.90)	1.22	(0.77–1.92)	1.20	(0.85–1.69)
Region						
Central	1		1		1	
Eastern	0.97	(0.66–1.44)	1.04	(0.76–1.42)	1.03	(0.81–1.31)
Northern	0.88	(0.57–1.35)	0.74	(0.50–1.09)	0.79	(0.59–1.05)
Western	0.99	(0.59–1.64)	0.87	(0.61–1.23)	0.92	(0.67–1.26)
Relationship to household head						
Head	1		1		1	
Spouse	1.08	(0.50–2.34)	0.93	(0.57–1.51)	0.92	(0.64–1.33)
Relative	1.25	(0.62–2.49)	0.55**	(0.37–0.82)	0.63**	(0.44–0.89)
Marital status						
Married	1		1		1	
Divorced/separated/never married	0.48	(0.23–1.01)	0.89	(0.49–1.63)	0.78	(0.52–1.17)
Widowed	0.94	(0.56–1.59)	1.08	(0.65–1.80)	1.05	(0.73–1.49)
Religion						
Catholic	1		1		1	
Anglican	0.94	(0.66–1.35)	1.20	(0.93–1.56)	1.12	(0.90–1.39)
Muslim	2.02**	(1.32–3.10)	1.92***	(1.37–2.71)	1.99***	(1.48–2.67)
Pentecostal	1.19	(0.58–2.48)	1.71**	(1.15–2.57)	1.60**	(1.12–2.28)
SDA and others	2.06	(0.99–4.27)	1.45	(0.76–2.75)	1.83*	(1.04–3.22)
Poverty status						
Poor	0.91	(0.63–1.33)	0.73	(0.52–1.03)	0.76*	(0.59–0.99)
Not poor	1		1		1	
Household major source of earnings						
Farming	1		1		1	
Wages	1.33	(0.93–1.89)	1.38*	(1.04–1.81)	1.35**	(1.08–1.68)
Remittances	1.46	(0.89–2.39)	1.27	(0.93–1.74)	1.32*	(1.02–1.70)
Learnt a trade or technical skill						
Yes	1.19	(0.82–1.72)	1.17	(0.87–1.58)	1.20	(0.94–1.52)
No	1		1		1	
Household owns bicycle						
Yes	1.50**	(1.10–2.03)	1.16	(0.89–1.52)	1.27*	(1.06–1.54)
No	1		1		1	
Was ill or injured during past 30 days						
Yes	1.30	(0.92–1.85)	1.78***	(1.33–2.38)	1.56***	(1.25–1.96)
No	1		1		1	
Ever smoked/smokes						
Yes	0.76	(0.54–1.06)	1.08	(0.77–1.53)	0.94	(0.74–1.20)
No	1		1		1	
Disabled						
No	1		1		1	
Yes	2.01***	(1.36–2.99)	1.44**	(1.12–1.86)	1.62***	(1.31–2.00)
Gender						
Men					1	
Women					1.97***	(1.45–2.69)
Observations	1,241		1,387		2,628	

OR=odds ratios; CI=confidence intervals; *p<0.05; **p<0.01; ***p<0.001.

For all older persons, reporting of NCDs was significantly associated with age group, household headship, religion, poverty status, and source of earnings, ownership of a bicycle, sickness in the last 30 days, disability, and gender. Reporting an NCD was positively associated with being a woman [odds ratio (OR)=1.97, 95% confidence interval (CI): 1.45–2.69], advanced age: 70–79 years (1.32, 1.02–1.72), household headship, being Muslim (1.99, 1.48–2.67), Pentecostal (1.60, 1.12–2.28), and being an SDA or other religion (1.83, 1.04–3.22). Being poor (0.76, 0.59–0.99), earning wages (1.35, 1.08–1.68), and receiving remittances (1.32, 1.02–1.70) were associated with NCDs among all older people. The odds of reporting an NCD were increased among older people who owned bicycles (1.27, 1.06–1.54), were sick in the last 30 days (1.56, 1.25–1.96), and disability (1.62, 1.31–2.00). However, region, marital status, having learnt a technical skill, and smoking history had no significant association with NCDs among all older people.

Among older men, reporting of NCDs was significantly associated with religion, poverty status, ownership of a bicycle, and disability. Muslim men were more likely (2.02, 1.32–3.10) to report NCDs compared to Catholics. Those who owned bicycles (1.50, 1.10–2.03) were more likely to report NCDs than those who did not. Men who reported disability (2.01, 1.36–2.99) were more likely to report NCDs than those who were not. Age group, region, household headship, marital status, wealth status, source of earnings, having learnt a technical skill, illness in 30 days, and smoking were not significantly associated with NCDs among older men.

For older women, self-reported NCDs were associated with age group, household headship, religion, poverty status, source of earnings, sickness in the last 30 days, and disability. Reporting NCDs was positively associated with age 60–69 years (1.44, 1.09–1.92), household headship, being Muslim (1.92, 1.37–2.71), and being Pentecostal (1.71, 1.15–2.57). Earning wages (1.38, 1.04–1.81) was positively associated with NCDs among women. Reporting an NCD was higher among women who were sick in the last 30 days (1.78, 1.33–2.38) and disabled (1.44, 1.12–1.86). Region, marital status, poverty status, having learnt a technical skill, owning bicycles, and smoking had no association with NCDs among all older people.

## Discussion

This article aimed at investigating the prevalence of and risk factors of self-reported NCDs among older Ugandans. From the analyses, 2 in 10 (23%) of the older Ugandans reported at least one of the three NCDs (hypertension, diabetes, and heart disease). This shows that NCDs are becoming an important cause of morbidity and mortality in Uganda, just like other low- or middle-income countries (3, 4, 27). A study in South Africa reported the prevalence of at least one NCD at 28% (4). The prevalence of hypertension ranged from 12 to 69% among garage workers to rich traditional chiefs in West Africa (28). In Tanzania, NCDs were estimated at 27% (29). Uganda's NCD prevalence, although not very high, could be under-reported.

Advanced age was associated with NCDs among older people. For example, the prevalence of hypertension rises with age. Some studies have reported similar findings in South Africa and Ghana (4, 7). Advanced age is associated with poor nutrition, reduced intake of fruits and vegetables among older persons (4, 7). In addition, advanced age is associated with low physical activity as a result of physical disability (7, 9), which increases the risk of hypertension.

Older persons who headed households reported NCDs more than those who were related to them. In Uganda, in the era of HIV/AIDS, older persons who head households are usually caregivers of orphaned grandchildren (30–32). These caregiving roles are associated with stress and vulnerability, on the part of those with advanced age (33). The ensuing stress related to household headship and its care giving roles poses a risk for depression and mental ill health, which predispose them to hypertension (34–36). A study in Nepal linked stress to cardiovascular disease (10).

Religious affiliation significantly predicted the risk of NCDs. Muslim, Pentecostal, and SDAs were more likely to report NCDs than Catholics. The risk of NCDs could be attributed to the sedentary living connected with business occupations that Muslims majorly engage in their lifetime (16) such as shop-keeping (6). The explanation for Pentecostals and SDAs is an area for further research.

Being poor was negatively associated with NCDs among older persons. NCDs were less prevalent among the poor older persons. This confirmed findings from a South African study (4) and a Ugandan study (27) where NCDs were associated with better socio-economic status. Socio-economic development is accompanied by lower physical activity and lifestyles that are more sedentary (7). In addition, higher incomes are also associated with affluent lifestyles including dietary modifications and obesity that are risk factors for hypertension (7, 28). Similarly, older persons who depended on wages and remittances had increased odds of reporting NCDs compared with those who depended on farming. Older persons who are farmers engage in physically taxing activities and healthier diets that lower the risk of NCDs (7, 28).

Older men and persons from households who had a bicycle were more likely to report NCDs than those who did not. This association was stronger among older men alone and all older persons and not for older women alone. We expected that the bicycle owners do physical exercise, which is protective of NCDs. However, this was not the case. The likely explanation could be that the bicycle owners have better access to health care (37), where they are able to know their health status better than those who do not hence the higher likelihood of reporting NCDs.

The likelihood of reporting NCDs was more likely among women and all older persons who were ill or sick during the last 30 days. This association was not significant for older men, perhaps due to the under-reporting of ill health or NCDs, among them compared to older women (4, 16). Consistently, disability was significantly associated with reporting of NCDs among men, women, and all older persons. Ill health and disability lead to physical inactivity and a sedentary lifestyle, which increase the risk for NCDs (4, 16, 38).

Finally, older women were more likely to report NCDs. Similar results have been observed in South Africa (4) and Ghana (7). Older women have more risk factors: smoking, alcohol, and insufficient fruits and vegetables intake, which predispose them to NCDs than older men (15, 16, 39). Other studies have advanced that women's susceptibility to NCDs is linked to physical inactivity and obesity, which are reported to be higher among women than the men (16). In addition, women have more access to health care throughout their life course than men (5). Owing to their constant interaction with the healthcare system in their lifetime, during maternity and as health caretakers, they tend to learn about their health conditions better than the men do.

### Strength and limitations of the study

This study provides the evidence base on the prevalence of NCDs and their associated risk factors among older people using a nationally representative sample. This is useful in planning and rolling out the NCDs initiatives by the Ministry of Health (MoH) in Uganda.

Despite the strength of the article, these findings should be interpreted with consideration of some limitations. First, the self-report of health conditions including NCDs (hypertension, heart disease, and diabetes) and disability is likely to be an underestimate of the actual prevalence (7). The UNHS data did not include other NCDs such as cancers and other chronic respiratory diseases. People tend to under-report poor health outcomes (4). For example, in Ghana, self-reported prevalence of hypertension was 14% and measured hypertension was 51% (7). In addition, under-reporting of unhealthy behaviours such as smoking may be due to social desirability biases (16) in Uganda.

Second, the cross-sectional nature of the UNHS data does not allow for proper ascribing of causality and associations between explanatory variables and NCDs. First, it is difficult to ascertain whether NCDs precede disability or disability precedes NCDs. In addition, it is difficult to monitor trends and patterns over time (4). Longitudinal data or cohort studies for older persons would be ideal to describe these associations.

Finally, the data were collected from older persons who were available or usual or regular household members during the survey. Older people in institutions (such as hospitals, prisons, or care homes) who had been away for more than 6 months were not included as the case was in the South African study (16).

## Conclusions

Self-reported NCDs were associated with advanced age, household headship, being a woman, being Muslim or Pentecostal or SDAs, being rich, being dependent on wages or remittances, being disabled, and ill in the last 30 days. NCDs were associated with men who were Muslim, owned bicycles, and reported disability. Among older women, NCDs were more among those who were advanced in age, headed households, were Muslims and Pentecostals, were dependent on wages, and were sick and disabled. Therefore, social and health inequalities exist among older people in Uganda.

The MoH needs to intervene by preventing and controlling NCDs in Uganda. The first approach is to create awareness about NCDs among the general public including older persons (16). Secondly, there is a need to scale up the supply of essential drugs for the treatment of NCDs including hypertension, diabetes, and heart diseases in public health facilities. Special attention should be given to older persons of advanced age, women, and those with physical disability.

Finally, there is a need for specialised longitudinal or surveillance studies of older people to monitor the trends and patterns of NCDs over time (4). This could help to capture better estimation of NCDs and understanding of the risk factors among older people for planning and policy purposes by the MoH. In addition, panel surveys would alleviate the limitations of cross-sectional studies in ascertaining causality and associations with NCDs and explore the influence of religion on NCDs. Such studies should address the risk factors of NCDs: physical activity, dietary intake, alcohol consumption, family history, overweight, or obesity among others (40).
